# Retraction Note: Circular RNA ITCH suppressed prostate cancer progression by increasing HOXB13 expression via spongy miR-17-5p

**DOI:** 10.1186/s12935-024-03344-y

**Published:** 2024-04-30

**Authors:** Xuegang Wang, Rong Wang, Zhun Wu, Peide Bai

**Affiliations:** 1 Department of Urology, The First Affiliated Hospital of Xiamen University, The First Clinical College of Fujian Medical University, Xiamen, Fujian 361003 People’s Republic of China; 2 Department of Urology, The Jintan Hospital Affiliated with Jiangsu University, Changzhou, Jiangsu 213200 People’s Republic of China

**Retraction Note: Cancer Cell International (2019) 19:328**

10.1186/s12935-019-0994-8

The Editors have retracted this article at the corresponding author’s request. After the publication of the article, image overlap concerns were raised. The corresponding author stated a third-party biological company was handling the data of this article. The authors did not store the data properly and they are unable to get the data from third-party now.

The Editors therefore have no longer confidence in the data presented in the article.

Image Concerns:

- Image overlap between Fig. 3C (DU145, NC) and Fig. 3C, KHYG-1 (LMPI + DMSO) of [1].
- Image overlap between Fig. 6C (LNCaP, CircITCH + miR-17-5p) and Fig. 6C (Hep3B, 2) of [2].
- Image overlap between Fig. 2D (DU145, NC, and circITCH) and Fig. 9B (last two images on the right side) of [3].

The corresponding author, Rong Wang has stated that all authors agree to this retraction.
